# Assessing Unmet Information Needs of Breast Cancer Survivors: Exploratory Study of Online Health Forums Using Text Classification and Retrieval

**DOI:** 10.2196/cancer.9050

**Published:** 2018-05-15

**Authors:** Susan McRoy, Majid Rastegar-Mojarad, Yanshan Wang, Kathryn J Ruddy, Tufia C Haddad, Hongfang Liu

**Affiliations:** ^1^ Department of Electrical Engineering and Computer Science University of Wisconsin-Milwaukee Milwaukee, WI United States; ^2^ Mayo Clinic Rochester, MN United States

**Keywords:** online health forum, automated content analysis, text retrieval, text classification

## Abstract

**Background:**

Patient education materials given to breast cancer survivors may not be a good fit for their information needs. Needs may change over time, be forgotten, or be misreported, for a variety of reasons. An automated content analysis of survivors' postings to online health forums can identify expressed information needs over a span of time and be repeated regularly at low cost. Identifying these unmet needs can guide improvements to existing education materials and the creation of new resources.

**Objective:**

The primary goals of this project are to assess the unmet information needs of breast cancer survivors from their own perspectives and to identify gaps between information needs and current education materials.

**Methods:**

This approach employs computational methods for content modeling and supervised text classification to data from online health forums to identify explicit and implicit requests for health-related information. Potential gaps between needs and education materials are identified using techniques from information retrieval.

**Results:**

We provide a new taxonomy for the classification of sentences in online health forum data. 260 postings from two online health forums were selected, yielding 4179 sentences for coding. After annotation of data and training alternative one-versus-others classifiers, a random forest-based approach achieved F1 scores from 66% (Other, dataset2) to 90% (Medical, dataset1) on the primary information types. 136 expressions of need were used to generate queries to indexed education materials. Upon examination of the best two pages retrieved for each query, 12% (17/136) of queries were found to have relevant content by all coders, and 33% (45/136) were judged to have relevant content by at least one.

**Conclusions:**

Text from online health forums can be analyzed effectively using automated methods. Our analysis confirms that breast cancer survivors have many information needs that are not covered by the written documents they typically receive, as our results suggest that at most a third of breast cancer survivors’ questions would be addressed by the materials currently provided to them.

## Introduction

### Study Objectives

Health concerns are prevalent among breast cancer survivors both during and after their cancer treatments. These health concerns are ongoing and can include topics such as symptoms and side-effects, fear of cancer recurrence, and coordination of follow-up cancer screening. As a result, breast cancer survivors have a wide range of emotional and information needs that will vary over time. These issues can have an impact on both a survivor’s quality of life and future decisions about health care. Medical providers need an accurate assessment of survivors’ information needs, especially regarding any unmet needs, in order to provide appropriate educational resources to improve quality of care and to support patients’ successful transition from treatment by an oncologist to care from a general physician and self-management.

The aim of this study is to assess the unmet information needs of breast cancer survivors [1-5] from the patient’s perspective and to develop methods that can be used to improve the information resources provided to them. However, the problem and the methods are not specific to cancer. There are two subtasks to assessing the problem of unmet information needs. The first task is identifying what information the population of concern perceives as necessary, but they feel has been inadequately addressed. The second task is determining whether the perception is due to a true gap in the resources that are being provided to them or to ineffectiveness in how information is being provided.

Determining the nature of any perceived information gaps would assist in directing efforts to appropriately address them. Some gaps can be addressed by adding more content, as long as the right content is added, and it can be located easily. One might also want to consider creating more accessible means of providing content, so the relevant information can be easily found. Most existing resources for survivors, which include brochures, books, and care plans, are static paper documents or webpages. By design, static content must balance the goals of covering the most commonly needed topics, while remaining manageable in size. Finding the required information can be difficult even when a resource provides it, because the relevant knowledge may be surrounded by less relevant information or may not be expressed in the terminology that a person expects. Voice assistants and chatbots that support question-answering could target dynamically expressed information needs, to eliminate searching, but they also require very specific information about the topics of interest and how they might be expressed. Assessing patients' perspectives on their unmet information needs, in the most authentic means possible, will assist in the design of new tools to address these problems.

This study will consider postings to peer-to-peer online health forums as a relevant resource for learning about patients’ unmet information needs because the very fact that a person posted an information-seeking question online is evidence of their perceived need. The postings also provide information about the language patients typically use to describe the information that they need. Using online forum data also allows for the assessment of needs over a wide span of time and from a diverse population that resides and receives care across a wide geographic area. We envision that the selected health forums could be accessed periodically to obtain up-to-date information about the needs of breast cancer survivors, and this information could be shared with content experts to guide them in creating and refining educational resources. Because these forums might contain information (posts) unrelated to information-seeking, automatic methods would be applied to discriminate true expressions of information need from similar sentences, such as questions that are primarily social or intended to clarify a previous statement. To obtain more specific information, sentences would be classified into meaningful categories and keywords or concepts extracted and subject to further analysis.

### Background

There have been several recent efforts to assess the unmet needs of cancer patients. Many use the Supportive Care Needs Survey [1-5]. This validated questionnaire covers 5 domains; namely psychological, health system and information, physical and daily living, patient care and support, and sexuality needs. The need for counseling to deal with psychological distress and the need for information about treatment, prognosis, wellness, and managing symptoms and side-effects have been the most commonly reported unmet needs in cancer patients. This survey and the results provide a useful starting point for an automated analysis.

Having multiple methods for assessing unmet information needs would be valuable, as relying only on survey results introduces bias that limits the reliability of the results. Bias can arise from how questions are worded, how subjects are recruited, and the beliefs and psychology of individual subjects when interacting with researchers or participating in a survey. The needs of an individual can also change over time. In our experience with developing a prototype phone-based question-answering tool, less than half the topics mentioned in surveys and focus groups of providers and clients were mentioned in the questions posed to the tool by subjects during an at-home user study and the subjects also asked many questions not previously identified [6].

Online health forums have been found to be a valuable resource for gaining the patients' perspective on their health concerns. As such, researchers have analyzed online forum data to learn about the experiences and needs of groups that might be difficult or sensitive to reach, including patients taking new medications [7] and people with eating disorders [8]. The results reveal evidence of unmet information needs including questions about indications and contraindications, proper use and storage, diet and drug restrictions, side effects, safety, and efficacy [7]. The importance of examining forum data is also supported by survey studies of health forum users, who report finding them to be valuable sources of health information and support, including both the active posters as well as “lurkers” (ie, those who read but do not post), which suggests that the forums are a place where participants return over time as new information needs arise [9].

The prior studies on health forums [7-9] all relied on a manual analysis of content that would be costly to replicate on a regular basis. A more automated method of analysis would be beneficial, but typical postings to online health forums, as shown in Textbox 1, have many characteristics that would present challenges to applying automatic approaches (for clarity, the sentences in the post have been separated and the general function of each sentence has been noted underneath in italics). A qualitative analysis of several forums for breast cancer survivors revealed a number of distinctive features. First, the vocabulary used to express information needs contains a mix of terminology from clinical medicine, consumer health, and daily living (including family, finances, and hygiene). Second, the style of interaction is often similar to semiformal written correspondence. For example, in addition to information exchange, the posts may include text that expresses social conventions, such as salutations and closings. However, sometimes the posts resemble text messages and forgo (or abbreviate) traditional conventions. The sequences of the posts are also similar to spoken conversations and involve turn-taking that shifts focus among the participants. Turns may address multiple functions including control of the dialogue (eg, to start a conversation or to invite the next person to give a response), to enhance a social relationship, or to provide or request specific information. The final feature found revealed that individual posts, and the sentences that comprise them, often vary greatly in length, possibly reflecting the variety of devices that people use to post online. In the longer posts, one often observes survivors sharing extensive information about their journeys, which both establishes a context for seeking information and creates a social connection to other survivors which encourages trust. Several sentences may be used to separately introduce a topic, provide context, and make an information request that includes references to the other sentences. The post shown in Textbox 1 is the start of a much longer conversation that overall contained 23 separate posts by different participants, with a total of 110 sentences.

The characteristics found in forum postings represent challenges for automated text classification because classification approaches generally work best when items in a class are similar to each other and each item of data has a unique class. To reduce the number of classes an item of data might represent, one can split posts into individual sentences. However, sometimes even short sentences can contain more than one class. Also, splitting the posts may make it necessary to later combine the results from separate sentences to fully understand a sentence. For example, in Textbox 1, to understand the query, “Anyone else have this difficulty,” one must refer to previous sentences to identify that “this difficulty” refers to the previously mentioned problem with a prosthesis used after a mastectomy being “hot and uncomfortable.”

### Approach

This study contributes both to the problem of identifying information needs survivors perceive as unmet and to the problem of identifying potential gaps in the knowledge commonly provided to them. This work involves four steps, namely (1) creating a taxonomy, (2) annotating sentences from two online health forums with categories from the taxonomy, (3) developing and evaluating classifiers using the annotated data, and (4) using an annotated corpus and information retrieval methods to measure the gap. Using any supervised classification approach requires having a corpus of annotated data and using two provides more generality. We developed a new taxonomy to annotate the data with categories related to the previously noted concerns of survivorship, including treatments and the physical and psychological problems afterwards, as well as categories related to the structure of posts, such as social or referential expressions.

Developing classifiers involves comparing several alternative algorithms and combinations of features for training classifiers using the annotated data. This step is necessary, because while there are a large number of different algorithms for building automated classifiers, there is no known method for predicting which algorithm, or which combination of possible input features, is best for a given problem.

**Example post to a health forum for breast cancer survivors. Each sentence in the post is presented on a separate line, with its general function as described in this study noted in italics below the sentence.**

Hi to all the women out there! I was diagnosed with breast cancer, stage 1, 11 months ago

*(Social greeting)*

I am 59 years old

*(Non-medical background)*

I had a right breast mastectomy and chose to not get breast reconstruction

*(Medical)*

The prosthesis I was given is hot and uncomfortable, so I am finding that I do not use it

*(Physical problem)*

Anyone else have this difficulty

*(Expresses an information need)*

I have recently moved and need to start all over with a new oncologist

*(Other problem)*

How do I choose one?

*(Expresses an information need)*

To find the best approach, one must systematically evaluate several different classification methods and input features, starting with the most basic features (words or pairs of words), and then assessing more complex ones, such as features that would capture lexical semantics or local context. We assess using topic models and word embeddings as a way of introducing semantic information that sometimes generalizes better than a simple word or bigram model. We also assess using the categories associated with the immediately preceding and immediately following sentences to capture some of the local context.

To measure the potential knowledge gap, we apply commonly used methods from information retrieval. This step serves two functions. First, it gives a better understanding of unmet needs. It also provides a way of assessing the usefulness of the data that one could collect from social media. The technique uses survivors’ own language as queries to an indexed set of commonly distributed documents. Annotators judged the relevance of the top-ranked results. If documents are retrieved that seem relevant, it would suggest that there is no knowledge gap, but there may be a problem with survivors not having the right document when they need it. If no document is retrieved that seems relevant, then there is likely a gap (but there could also be a difference in language that would make both search and understanding the document difficult.) Both problems would warrant further review.

Thus, the four steps of this work, together, will reveal the extent to which automatic approaches can identify expressions of unmet information need from online health forum text and provide new information about how resources might need to be improved.

## Methods

### Data collection

This study used data collected from two online health forums. We started by creating a data set from a MayoConnect (MC) forum for breast cancer survivors. This forum was selected primarily because of its local interest. It also included data spanning at least five years, had active postings, and (at the time) was a peer-to-peer forum. To create a data set, we extracted the complete set of conversations available at the time, each consisting of multiple posts from different authors, removed any metadata and split the sentences into sentence-type units using an automated procedure. This data set consists of 65 conversations which yielded 1943 items for coding. The average number of sentences in a post was 6.35 (SD 4.42), the average number of words per sentence was 14.04 (SD 6.30) and the average number of characters per word was 5.24 (SD 2.31).

To better assess the generality of any findings, a second forum, the American Cancer Society Cancer Survivors Network on breast cancer (CSN), was selected. It also had active posts spanning at least five years and involved peer-to-peer interaction. This forum met the additional criterion that the forum is easily accessible for research. One can download archived posts using open-source software called curl [10]. From this site we created two data sets; one that contains complete conversations and is similar in size to the MC data set, which is henceforth referred to as “CSN,” and another, smaller data set of randomly selected sentences, “CSN-R.” To select conversations for the larger dataset from CSN, a simple rule-based classifier was created to identify sentences that might represent an expression of an information need and only used conversations that contained at least one such sentence. We used this strategy in the hopes of increasing the density of information-seeking examples in the data set, as the natural density appeared to be less than 5%, which might affect the coders.

To build the rule-based classifier, a training set was made from the complete annotated MC data set and a small sample (<200 sentences) from the CSN forum data which was labelled separately before extracting the complete CSN data set. This yielded about 150 positive examples of information needed. Additionally, a vocabulary (V) was defined which contained potential question cues or components of one, such as auxiliary verbs, pronouns, wh-words (when, where, why, and how), and verbs, nouns, and adjectives associated with direct and indirect requests for information (wondering, wanting, trouble, anyone, similar). The patterns for the rules were then created using the algorithm shown in Textbox 2.

This simple classifier is helpful in building a data set but both false negatives and false positives will occur due to unseen examples and counter examples. To investigate whether this classifier would be more broadly useful, a test set, CSN-R, was created for evaluating both the rule-based classifier and the statistically trained classifier for expressions of information need which is planned for development. CSN-R (the test set) comprised a random sample of 1000 sentences extracted from the CSN forum for a time period that, importantly, did not overlap with the other larger sample.

For the main CSN dataset, 195 conversations were obtained which were then split into posts and then the posts were further separated into sentences. From this process 2246 items that could be coded were obtained. The average number of sentences per post was 11.52 (SD 5.29), the average number of words per sentence was 13.97 (SD 5.30), and the average number of characters per word was 6.12 (SD 3.21).

**Iterative algorithm for building patterns of expressions of information need.**

For each positive example of the training set

If the example is not already matched by a pattern,

then generate the smallest set of bigrams from V such that

the positive training example has all the bigrams in the set

and no negative training example has all the bigrams in the set

### Taxonomy for Supervised Classification

Following a review of the literature related to past assessments of the needs of breast cancer survivors [1-5], and some prior taxonomies [11,12], a new taxonomy was iteratively developed. As mentioned above, prior survey work [1-5] revealed that the most commonly reported unmet needs in cancer survivors include psychological distress and the need for information, especially about treatment, prognosis, wellness, and managing symptoms and side-effects. Some categories included in the Supportive Care Needs Survey, such as relationships or sexuality, were considered but not included in our taxonomy because they did not occur in our data. In examining prior taxonomies, some relevant categories were found, such as expressing an information need, or providing medical background, but also many categories rarely mentioned by survivors, including anatomy, causes, complications, diagnoses, manifestations, and susceptibility. Prior taxonomies lacked categories for treatments and for physical or psychological problems associated with survivorship. Furthermore, it was found that the topics and information need were orthogonal types, suggesting it would be prudent to use separate categories that could be later combined.

The new taxonomy includes a single binary category “has information need” or “HASN” to indicate whether an entity expressed an information need. This category covers both direct questions as well as implicit questions expressed as statements, such as “I am concerned about...” or “I was wondering about...” It also includes 10 categories to indicate the primary type of information provided or requested, namely medical, resource, social, psychological, background, wellness, physical, previous, other, and multiple. These primary types of information correspond to medical events (“medical” eg, clinical observations, diagnoses, and interventions), educational resources (“resource” eg, books or websites), social interaction (“social” eg, greetings, invitations to talk, thanks, or good wishes), self-identified psychological problems (“psychological” eg, fear or sadness), non-medical personal information (“background” eg, age, family, or employment), wellness tips (“wellness” eg, diet, hygiene), and self-identified physical problems (“physical” eg, pain, hair loss). The other categories are used for exceptions where appropriate. “Previous” is used when the main topic of a sentence requires interpreting a referring expression to another sentence; “other” is used for information topics that fall outside the realm of any of the defined topics (such as travel); and “multiple,” used only for coding the MC data, is used to indicate when a sentence covered multiple categories. The category “multiple” does not apply to the CSN data as when coding the data, annotators were allowed to specify up to two information categories and did not explicitly label data as “multiple.” The complete annotation guideline, along with examples, is given in Multimedia Appendix 1.

### Data Annotation

Four people performed the data annotation tasks. The team included 2 experts from the research team who had expertise in computer science and 2 nurse abstractors with experience in data abstraction for health sciences research. The 2 experts developed the written guidelines for annotation. The nurse abstractors were trained to conduct data annotation using a small sample set (approximately 200 items). For the MC data set, one expert and one trained abstractor independently annotated the data, and another expert adjudicated the results. For the CSN data set, one trained abstractor and one expert independently coded the data, and another trained abstractor adjudicated the results. For the CSN-R dataset, two experts annotated the data and one trained abstractor adjudicated the results. For rating the relevance of retrieved educational documents, the same 2 trained abstractors acted as independent judges.

Inter-annotator agreement was assessed for each class separately using both simple counts and the percentage of the final calculated quantity of the class captured by the agreed items. This measure was used as the sample sizes were quite variable and often small. Using this measure, for the information classes (eg, “medical,” “resource,” “social,” and “psychological”) the agreed items for the MC data set covered from 11% (16/147, “previous”) to 96% (541/597, “medical”) of the total number of items determined to be in each class. For the CSN data set, the agreed items covered from 62% (454/728, “other”) to 100% (32/32, “resource”) of the final items in each class. Using this same measure, the agreement for the class “has information need” covered 20% (22/110) of the final items for the MC data set, 69% (135/196) for the CSN data set and 66% (23/35) for the CSN-R data set. The agreement counts for all categories across all data sets are provided in Multimedia Appendix 2. After the annotation of the MC data set, the guidelines were revised so that annotators could assign multiple categories to each sentence and therefore the category “multiple” was no longer used. Additionally, the description of the protocol for coding “has information need” was improved to better capture indirect expressions of information need, which had been frequently missed.

### Assessment of Information Needs and Content Expressed in Social Media

To assess how well these automatic approaches to analyzing social media text can identify expressions of unmet information needs and help to identify the nature of the need, we performed 3 studies using the annotated data sets, exploring several alternative forms of semantic and statistical analysis. The first two studies consider the distribution of sentences across categories of the taxonomy and the types of semantic information expressed in the sentences. The third involved experiments training classifiers with different algorithms and features using the annotated data.

#### Analysis of Distribution of Sentences across Categories of Taxonomy

After annotation of the complete MC data set, inter-annotator agreement was assessed and any differences between the annotations were adjudicated using an additional annotator and some discussion. The distribution of sentences across each of the categories of the taxonomy and the distribution of categories for sentences marked as indicating having a need was calculated.

#### Content Analysis of Social Media

Using the MC and CSN data sets described above, we identified the concepts most closely associated with each of the annotated and adjudicated categories, using MetaMap [13]. The concepts were selected by counting the number of occurrences of each concept in the sentences associated with each category and ranking them based on the size of those counts.

#### Assessment of Information Needs Using Text Classification

We trained and tested Naïve Bayes, linear Support Vector Machines, and Random Forest (RF) classifiers for each of the information classes (“medical,” “social,” “psychological,” “background,” “wellness,” “physical,” “previous,” and “other”) as one-versus-the-others using 10-fold cross-validation as implemented in Weka (machine learning software) [14]. Thus, if uniform distribution of the categories across the folds is assumed, in each iteration, the number of positive examples for each class ranges from approximately 55-535 from a total of 1750, depending on the class. We evaluated the following input features: words and bigrams alone, words and bigrams along with features derived from topic modelling, words and bigrams along with features derived from word embeddings, and words and bigrams along with features to represent the local context. For each combination, the precision, recall and F-measure using the functions that Weka provides were computed.

For the topic-modelling features, latent Dirichlet allocation [15] was used to generate sets of words corresponding to different topics appearing in the sentences of the posting. We used the MC data set, which contains 1943 sentences, with an average length of approximately 14 words. For each topic, a feature corresponding to the probability that the sentence contained that topic was added. This is calculated as the percentage of the tokens in the sentence generated by a topic. We used 50 topics, each of which corresponded to 15 words. To determine the number and size of topics to use, we experimented with different numbers of topics (5, 10, 50, and 100) and different numbers of words (5, 15, 20, and 50) per topic, with the goal of creating topics, that upon manual inspection, appeared most coherent. For our data, 50 topics with 15 words per topics appeared best. Some examples of these topics are shown in Textbox 3. The topics shown appear to correspond to medical treatments and tests, family and friends, and parts of social greetings.

For word embeddings, pretrained word vectors generated by the GloVe algorithm were used [16]. The training corpus contains Wikipedia and Gigaword (newswire) text. To use word embeddings as features, the deepLearning4Java library and GloVe pre-trained word vectors corpus with 50 dimensions were used. We generated vectors for all words in each sentence of a forum posting, calculated the average of the vectors and used the average vector to add features for the classifier, such that each element of the average vector adds one feature in the classifier for each sentence.

For the local context features, we added binary values for each of the information types using the values determined from the hand-labelled results for the immediately preceding and the immediately following sentences.

After determining the best classifier (RF) and best set of features (words, bigrams, and local context) using the MC data set, we trained and tested on the annotated CSN dataset using 10-fold cross validation with the same combination of features and analyzed the results using standard measures of precision, recall and F-measure. The feasibility of training on data from one forum and using it to classify data from another forum was also assessed.

Finally, potential classifiers for identifying sentences that express an information need were evaluated using a small test set of randomly selected and hand-annotated sentences, CSN-R, that had not been used for any other purpose. Both the rule-based classifier and three different statistical learning models (Naïve Bayes, linear Support Vector Machines, and RF) were evaluated. The statistical classifiers were trained with the combined hand-annotated data from the MC and the CSN data sets using only words and bigrams as features and the precision, recall, and F-measure were computed.

### Assessment of Knowledge Coverage using Text Retrieval

To review the adequacy of current patient education materials, we performed the following steps:

Electronic copies of brochures typically given to breast cancer patients at the Mayo Clinic Breast Center were obtained and each page was indexed separately using Elasticsearch [17], an enterprise search engine. Complete pages, rather than sentences or subsections, were indexed because we did not want to overestimate a gap if the query terms spanned multiple such units.

A set of 136 queries, based on our hand-coded results from the CSN dataset, was created. Hand-annotated data was used so that we would not over-estimate the gap; however, the ultimate goal would be to perform similar reviews using sentences that had been classified using an automated process.

**Sample topics derived by latent Dirichlet allocation processing (w: word).**

topic:23

w1:chemo w2:treatment w3:surgery w4:pain w5:mastectomy w6:treatments w7:rads w8:lumpectomy w9:results w10:reconstruction w11:tumor w12:scan w13:bone w14:test w15:biopsy

topic:41

w1:family w2:someone w3:husband w4:friend w5:friends w6:talk w7:mom w8:sister w9:daughter w10:sisters w11:small w12:couple w13:together w14:mother w15:kids

topic:42

w1:hugs w2:thank w3:read w4:thoughts w5:wish w6:lots w7:sending w8:questions w9:enjoy w10:welcome w11:wishes w12:question w13:answer w14:send w15:sent

We started with all the sentences marked as “has information need.” Then, we manually removed any duplicates, where a sentence was defined as a duplicate if it had been marked as “previous” and immediately followed another sentence marked as “has information need.” For example, in the sentence pair *“I am concerned about whether insurance companies cover this like they do taxol. Any answers out there?* ” the second sentence would have been removed. For the remaining sentences classified with an information category (not “previous”), stop-words were removed, the tf-idf score for each remaining content term was computed, and up to ten of the highest-scoring terms were selected. For sentences classified as referential (“previous”) but not considered duplicates, we obtained, scored, and selected up to ten content terms from the nearest sentence with a nonsocial information category. For each query, we then used the search engine to obtain a ranked list of documents. The ranking was based on the standard similarity algorithm provided by Elasticsearch, Okapi BM25, which accounts for term frequency and inverse document frequency.

A formatted file was created to show the complete posting, the (highlighted) query and the two top-ranked, retrieved documents with matched portions also highlighted. More than two were not provided, because an examination of preliminary results did not reveal any cases where lower-ranked documents appeared relevant. Multiple raters were asked to specify, for each document, whether or not they felt that it satisfies the information need.

Simple agreement among judgements, not adjusted for chance, were computed and assessed overall coverage.

## Results

### Distribution of Sentences Across Categories

In the MC data, there were 65 conversations, which yielded 1943 sentences (Table 1). Among the sentences, 5.7% (110/1943) were identified as having an expression of an information need (HASN). In the CSN data set, there were 195 conversations, yielding 2246 sentences (Table 1). Among these sentences, 8.7% (196/2246) were identified as expressing an information need. In a smaller, randomly selected set of 1000 sentences from the CSN (CSN-R), 3.5% (35/1000) were information seeking questions.

The distribution of sentences among the categories identified above in both the MC and CSN data sets is shown in Table 1. In the MC data set, the distribution of sentences among the categories ranged from 3% to 31%, with the “medical” category being the most common (597/1943, 30.7%) and the “social” category being the second most common (353/1943, 18.2%). Mentions of “psychological” and “physical” problems together accounted for a combined 11.7% (228/1943) of the sentences. Sentences most likely to discuss solutions (eg, “wellness” and “resource”) accounted for a combined 9% (175/1943) of sentences. In the CSN data set, the distribution of sentences among the categories ranged from 1% to 32% with the “other” category being the most common (728/2246, 32.4%), followed by the “medical” (473/2246, 21.5%), and “social” (443/2246, 19.7%). Mentions of psychological and physical problems accounted for a combined 11.4% (256/2246) of the sentences. Sentences potentially discussing a solution (“wellness” and “resource”) accounted for a combined 4.9% (110/2246) of the sentences.

The distribution of categories in the subset of sentences expressing information need is shown in Table 1. The most common information type for the identified information needs in MC data was “medical” (34/110, 31%). Upon manual inspection, we found sentences desiring information about interventions such as chemotherapy, radiation, reconstruction, or double mastectomy (17 sentences); information about outcomes such as chance of recurrence, spread of cancer, or general prognoses (9 sentences); information about diagnoses, such as being Stage 3, triple negative, or metastatic (6 sentences) and information about tests, such as value of biopsy, mammograms, and other tests (3 sentences). The second most common information needs involved physical problems, including soreness, (being) tired, or (having) hair loss, swelling, trouble swallowing, blood pressure spikes, breast pain, or bowel issues. “Resource” requests accounted for 8.2% (9/110) of information needs, “wellness” accounted for 2.7% (3/110), and “other” accounted for 7.3% (8/110). The remaining 34.5% (38/110) were marked as “previous,” indicating they contained references that needed context outside the sentence for their interpretation.

In the CSN data, “medical” was again the most common information type among the sentences expressing an information need (48/196, 24.5%), followed by physical problems. “Resource” requests accounted for 4.6% (9/196) of information needs and information about “wellness” and psychological problems accounted for 2.6% (5/196) each. Twelve percent (24/196) were marked as “other” and 41.3% (81/196) were marked as needing context outside the sentence for their interpretation.

### Content Analysis Across Categories

Content analysis presents an automated method for analyzing the content. The analyses of concepts detected by MetaMap are shown in Table 2, where the concepts are listed in decreasing order of frequency from most frequent to least. In the sentences expressing an information need in the MC data, the most frequently mentioned topics include “*side effects,*” “*surgery,*” and “*chemo*” and in the CSN data the most common topics included “*chemo,*” “*treatment,*” and “*normal.*” Across both, the general concepts “*Help,*” “*Look,*” and “*Experience,*” were also commonly mentioned, but these likely reflect the expression of need itself (eg, “*Looking for...”* or “*anyone with that experience*”) In non-need sentences, the most commonly mentioned MetaMap concepts included “*cancer,” “breast cancer,*” and “*chemo,*” and many more general words, such as “*years,*” “*now,*” “*take,*” “*good,*” and “*feel.*”

The concepts determined by MetaMap to be associated with the information categories in the MC and CSN data sets are also shown in Table 2. Overall, the most common concepts found in the “medical” category included the diagnoses (*cancer, breast cancer, diagnosed*) and interventions (*chemo, radiation, Taxol, treatment*). The most common concepts in the category for physical problems include *hair (loss), pain,* and *back (pain)*, as well as language to express their concern (*side-effects, issue, feel*). The most common concepts found in the category for psychological problems include *depressed, scared,* and *cry* and language to express the concern (*feel*). Overall, none of these concepts seem surprising and one might expect that typical educational materials might cover them well.

### Classifier Training Across Categories

After training and testing multiple classifiers and combinations of features, it was found that the best configuration used RF classifiers using words, bigrams, and local context-based features corresponding to the information labels of adjacent sentences. Table 3 shows the performance of the RF algorithm trained with and without local context features for the MC and CSN data sets, where the classifiers within each data set were trained and tested using 10-fold cross-validation. The performance of alternative classifier training algorithms (ie, Naïve Bayes and linear Support Vector Machines) and the addition of features from topic modelling and word embedding were also assessed but they were found to not be helpful and impaired the performance of classifiers across every category (Multimedia Appendix 3). When RF classifiers using local context features and trained on data from one forum but tested on another were considered, it was found that the performance was impaired for all categories, although this reduction was somewhat less for the medical and social categories (Table 4).

The results of evaluating the developed rule-based classifier and different learning models for a binary statistical classifier to identify sentences that express an information need using the CSN-R data set showed that a classifier trained using the RF algorithm was the most successful. The results for the statistical classifiers are shown in Table 5. The RF algorithm achieved a precision of .62, recall of .65, and F-measure of .63.

**Table 1:** Distribution of expressions of information need (HASN) and categories in the MayoConnect (MC) and Cancer Survivor’s Network (CSN) data sets.

Category	MC total, n (%)	MC HASN, n (%)	CSN total, n (%)	CSN HASN, n (%)
Any	1943	110 (6%)	2246	196 (8%)
Medical	597 (31%)	34 (31%)	473 (21%)	48 (24%)
Resource	87 (4%)	9 (8%)	32 (1%)	9 (4%)
Social	353 (18%)	0 (0%)	443 (20%)	9 (4%)
Psychological	61 (3%)	0 (0%)	63 (2%)	5 (2%)
Background	69 (4%)	0 (0%)	38 (1%)	0 (0%)
Wellness	88 (5%)	3 (3%)	78 (3%)	5 (2%)
Physical	167 (9%)	18 (16%)	193 (8%)	15 (7%)
Previous	147 (8%)	38 (35%)	425 (18%)	81 (41%)
Other	313 (16%)	8 (7%)	728 (32%)	24 (12%)
Multiple	60 (3%)	0 (0%)	N/A^a^	N/A

^a^N/A: not applicable. This category was not used when annotating the CSN data set.

**Table 2:** Five most frequent concepts for each information and topic category

Category	Top 5 MC^a^ concepts	Top 5 CSN^b^ concepts
Information need	experience, side effects, look, surgery, chemo	help, chemo, treatment, normal, experience
No information need	cancer, breast cancer, chemo, years, now	now, take, good, chemo, feel
Medical	chemo, cancer, radiation, breast cancer, diagnosed	chemo, radiation, now, taxol, treatment
Social	thank, hope, good, luck, best	Hi, thank, good, love, take
Psychological	feel, make, right, better, depressed	scared, go, feel, cry, thing
Background	live, years, breast cancer, now, old	years, old breast cancer, diagnosed, age
Wellness	help, shampoo, started, make, work	exercise, eat, help, diet, keep
Physical	hair, pain, back, side effect, issue	pain, back, hair, feel, Taxol
Previous	help, need, experience, make see	one, help, think, out, now
Resource	website, research, mayo, cancer, breast cancer	book, breast cancer, insurance, groups, site
Other	one, out, need, go, cancer	make, think, thing, out, feel

^a^MC: MayoConnect.

^b^CSN: Cancer Survivor’s Network.

**Table 3:** The performance of Random Forest classifiers for each category for MayoConnect (MC) and Cancer Survivor’s Network (CSN) data.

Category	MC data						CSN data					
	Without local context features			With local context features			Without local context features			With local context features		
	Prec^a^	Recall	F-measure	Prec	Recall	F-measure	Prec	Recall	F-measure	Prec	Recall	F-measure
Medical	.74	.73	.73	.90	0.91	.90	.65	.64	.64	.78	.75	.76
Social	.78	.78	.78	.85	.85	.85	.71	.70	.70	.83	.82	.82
Psychological	.73	.72	.72	.77	.76	.76	.69	.68	.68	.73	.74	.73
Background	.77	.77	.77	.77	.77	.77	.73	.73	.73	.74	.74	.74
Wellness	.76	.75	.75	.80	.79	.79	.67	.66	.66	.70	.71	.70
Physical	.80	.79	.79	.82	.83	.83	.64	.64	.64	.70	.70	.70
Previous	.61	.61	.61	.58	.58	.58	.70	.70	.70	.71	.71	.71
Other	.59	.59	.59	.84	.86	.85	.61	.60	.60	.67	.66	.66

^a^Prec: precision.

**Table 4:** The performance of Random Forest classifiers for each category tested on MayoConnect (MC) and Cancer Survivor’s Network (CSN) data and trained on either MC or CSN data, using local context features.

Category	Test MC data						Test CSN data					
	Train MC data			Train CSN data			Train CSN data			Train MC data		
	Prec^a^	Recall	F-measure	Prec	Recall	F-measure	Prec	Recall	F-measure	Prec	Recall	F-measure
Medical	.90	.91	.90	.71	.71	.71	.78	.75	.76	.71	.67	.68
Social	.85	.85	.85	.61	.69	.66	.83	.82	.82	.75	.77	.76
Psychological	.77	.76	.76	.51	.55	.51	.73	.74	.73	.50	.54	.52
Background	.77	.77	.77	.51	.53	.51	.74	.74	.74	.50	.52	.51
Wellness	.80	.79	.79	.55	.59	.56	.70	.71	.70	.51	.60	.55
Physical	.82	.83	.83	.56	.54	.55	.70	.70	.70	.60	.66	.63
Previous	.58	.58	.58	.54	.60	.57	.71	.71	.71	.55	.58	.56
Other	.84	.86	.85	.65	.56	.60	.67	.66	.66	.61	.63	.62

^a^Prec: precision.

**Table 5:** Results of classifier training to identify sentences expressing information need in CSN-R (data set of randomly selected sentences from the Cancer Survivor’s Network data set).

Learning model	Precision	Recall	F-measure
Naïve Bayes	.57	.75	.59
Random forest	.62	.65	.63
Support Vector Machines	.58	.71	.61

By contrast, the rule-based classifier achieved a precision of .43, recall of .26, and F-measure of .33. Upon closer inspection, it was determined that most false negatives (24/25) represented entirely new patterns (one was due to a misspelling of a word) and the false positives mostly represented unseen counter examples (eg, the bigram *how long* used adverbially rather than as a question cue).

### Assessment of Potential Knowledge Gaps

The two most highly ranked documents (N=272) for each of the 136 queries were assessed by two raters. Of the 136 queries, 33.1% (45/136) were found to have relevant content by at least one rater and 12.5% (17/136) were found to have relevant content by all raters. The agreement, calculated over documents, was 86.8% (236/272). One rater found that 10.3% (28/272) of the documents were relevant, while another rater found that 13.2% (36/272) were relevant. On the agreed items, 15 were annotated as relevant by both and 221 were marked as irrelevant by both. On 36 items, one annotator marked an item as relevant while the other marked it as irrelevant. In Multimedia Appendix 4, several example queries, as well as the page that was returned and how it was rated, are provided to illustrate cases where there is no gap and where there is likely a gap.

## Discussion

### Principal Findings

This study investigates methods to automatically identify the information needs of breast cancer survivors based on their postings to online health forums. We found that an automated content analysis using MetaMap provided information very similar to what we had observed and counted manually.

The classifier results were also promising and suggest that such an approach should incorporate some discourse-level analysis because many conversations in online forums cannot fully be understood without it. In the MC data set, it was determined that 34.5% (38/110) of the sentences that expressed an information need had a discourse-dependent aspect and, in the CSN, there was an even higher proportion of information needs expressed that depended on other sentences (81/196, 41.3%). Although this study focuses on the problem primarily from an individual sentence perspective, the results illustrate the potential value of adding information features from nearby sentences. When using only the words or bigrams as features, the F-measure did not exceed 75%. However, when features corresponding to the immediately preceding and following categories were added, F_1_ scores of 90% on “medical” and 83% on “physical” for MC and 82% on the “social” category in CSN (with 75% on the “medical” and 69% on the “physical”) were achieved.

The value of adding additional semantic features is less certain. When additional features based on topic modelling and word embeddings were added for training classifiers of information topics, it was found that instead of improving the analysis, it reduced the overall accuracy. We suspect that the sentences in online forums are too short, and the vocabulary is too heterogeneous, to benefit from topics or embeddings pretrained from more homogeneous corpora such as Wikipedia or newswire text. Indeed, when classifiers were trained on data from one health forum and tested on the other, it was found that the F_1_ values were uniformly lower than when data was trained and tested within the same forum.

We found that classifiers could also be used for identifying sentences that express an information need. The most successful approach in this study involved training a RF classifier, for which a precision of .62, recall of .65, and F_1_ of .63 on unseen test data was obtained. For comparison, a simple rule-based classifier was created for filtering, and it did much worse. This result is promising, and one might improve it by incorporating local context information.

The assessment of the gap between the expressed information needs and typical educational literature was revealing. Considering the results from our content analysis, none of the concepts mentioned in sentences expressing information need seemed surprising. Typical tests, procedures, and medications were mentioned, however, the results from our experiment using standard information retrieval techniques suggest that, at best, only a third of breast cancer survivors’ questions would be addressed by the materials currently provided to them, and at worst only one in eight.

In many of the matches found, the query sentence includes specific clinical language and the topic is somewhat expected (eg, mentioning a specific drug and whether it is normal to have a known side effect). There also tended to be a match when a general word was used in a very predictable way, for example support for survivors. Many failures to match seem like true gaps. Mismatches tended to occur when a question mentions clinical but common terms associated with breast cancer treatment (surgery, chemotherapy, or oncologists) but asks something uncommon or perhaps is considered too dependent on medical history (such as the prognosis after treatment). In this case, the raters felt the retrieved document, which provided only general information about going to see an oncologist for follow-up care, was not sufficiently relevant. Another gap was revealed when the information need query was about abnormal sensations after surgery and the retrieved information document discussed breast MRIs and what happens if the results are abnormal; this type of partial match is typical of an information retrieval approach. One interesting example of a query that was nearly matched mentions the terms *chemotherapy*, *Taxol*, and *hair* and asks when hair might regrow; however, the information page returned explains that hair loss is a common side-effect, but it only suggests how to cope with the side effect and no information on how long the problem might persist is provided. These results suggest that it would be valuable for information providers and health educators to know more about the specific questions cancer survivors or their friends and family are asking.

### Limitations

One limitation of this study is that the taxonomy for categorizing the forum sentences was generated based on a manual process. In addition, some of the annotation was done by people who helped develop the taxonomy, creating a possibility for bias. To reduce this risk, when the sentences were annotated, there was always one annotator or adjudicator involved who had not been involved in creating the taxonomy.

The accuracy for categories with fewer examples is lower than for those with more, which is typical for this approach. The accuracy achieved for the CSN data set was also generally slightly lower than for the MC data set. We suspect that this difference reflects the broader scope of nonmedical, physical, and psychological topics present in the CSN data set (with many more marked as “other”) and a higher degree of complexity in the posts. In fact, it was found that the individual posts in the CSN data were nearly twice as long as in the MC data. We also note that the CSN sentences also included more referring expressions. In this case, additional features, if carefully chosen, might improve classification accuracy. Here, the focus was on word-based features (unigrams and bigrams) and the information categories of nearby sentences. Experiments using topic models as features did not reveal them to be helpful, however the training set used for generating the topics was fairly small, which may have negatively affected the quality and effects of the topic features. We did not perform named entity recognition, such as for names of specific drugs or treatments for cancer, but we suspect that might have been helpful to improve accuracy.

A rule-based classifier was created with the goal of helping to select conversations for annotation, with the aim that it might have broader utility. Currently this approach performs poorly compared to using hand-labelled data to train a statistical classifier. Having a rule-based classifier, however, was useful before enough data is obtained to train a statistical classifier. The classifier increased the frequency of sentences expressing information need in our data from the expected rate of approximately 3% occurring in a random sample to approximately 6%. Furthermore, the increased concentration of sentences expressing information need may have been helpful in improving data quality, as we found that with a very low density in our random sample, the annotators seemed to miss more positive examples than in the earlier, larger data set. However, this result may also be explained as by selecting random sentences, background information was lost, and this was crucial in helping them recognize a need.

While the hand-labelled data was used for the local context features for classifiers, in a production system this would not be feasible. Instead, one could classify sentences sequentially (and just use the immediately preceding class) or one could train a sequence-based classifier, such as one based on Linear-Chain Conditional Random Fields [18,19].

During the assessment of knowledge gaps in the educational literature, we used the words from the expressions of need and, when a sentence was classified as referential, we added words from nearby sentences. This approach is reasonable for document retrieval, but not sufficient for question answering. We did not augment any queries with synonyms, as our raters (and the general public) would not necessarily know when two specialized medical terms, or a medical and a consumer term, are synonyms. As a result, the approach used may overestimate knowledge gaps because the desired content might exist but use a different term than the one in the query. Nevertheless, this approach is valuable as it provides a good indicator of the difficulty that people would experience in trying to address their information needs with the available educational literature. A domain expert, familiar with the literature distributed to patients, could take the information we provide to either verify the information need to create new resources, or to revise the existing resources so that needed information would be easier to find.

### Comparisons with Prior Work

Other researchers have explored methods to classify sentences in various online forums and other online short texts. Most past studies of online health communities [7-9] have used social scientific approaches that involve examining relatively small samples of data and identifying themes by manual coding. These studies, while they provide valuable insights, cannot easily be repeated for different forums or different points in time. These studies also use the entire message as the unit of analysis, which makes the coded data created unsuitable for automated methods of text analysis. Automated methods work best when units of analysis can be assigned a single or small number of labels. However, postings to Web-based health forums and internet email discussion groups, which are asynchronous and do not significantly limit the length of postings, tend to combine social communication with “technical information about treatments, side effects, clinical trials, empathic comments, requests for information, (and) meta-comments about group processes [20],” each of which will naturally involve a distinct sublanguage.

Zhang et al reports the use of automated classification methods for health forum posts [21]. In this study supervised machine learning methods are used to label the posts with the writer's (broad) intent. Two key differences between this work and our own have been identified, namely (1) they classify groups of sentences as a unit, ignoring their internal structure and (2) the classes seem more pertinent to new diagnoses than to survivorship. Specifically, the classifications are “Manage” (prevention, treatment options, and management of chronic illness), “Cause” (diagnosis of physical findings and test results), “Adverse” (negative side effects of treatments), “Combo” (multiple intents), and “Story” (social narrative and personal story-telling). They found that a simple word-based classifier performed poorly, with a precision at most of 62%, but that by defining and using new pattern-based features, a precision of 75% could be achieved. The new features included short sequences of lower cased and stemmed words, part-of-speech tags, and semantic groups from the Unified Medical Language System.

There have been four efforts to develop automated methods to assess the content of online health question-answering and health-related search data [11-12,22-23]. The data used in these studies differs from online health forums in that they do not include on-going dialogs (instead they are isolated attempts to receive an answer or search result) and they do not involve communication among peers, and thus lack many of the social aspects found in health forums (such as self-introductions or offers of support). This work, however, demonstrates the feasibility and some of the challenges of using automated methods for complex questions, which can be indirect (eg*, I would like to learn more about this condition*) or involve coordination (eg, *I would like to learn more about this condition and what the prognosis is for a baby born with it*). McRoy et al [11] examined questions sampled from several community question-answering Web sites. They developed a more fine-grained taxonomy than the one used here and methods to classify the specific type of information being requested, such as “definition”, “entity”, “explanation”, “property value”, “reference”, “diagnosis”, ‘outcome”, or “recommendation”, which would be useful for formulating a response, but is not needed for information retrieval. Roberts et al [12,23] have developed methods related to understanding consumer health questions submitted to the Genetic and Rare Diseases Information Center (GARD) website. For example, they have developed a taxonomy of different types of medical questions about a wide range of diseases [12] and methods for decomposing multi-sentence, multifaceted questions by classifying sentences as either a “question,” “background,” “coordination,” “exemplification,” or “ignore.” They also developed methods for identifying the disease of central concern, which might occur in any part of the question [23]. Phan et al [22] explored the use of topic modelling as a feature for the classification of short texts where the topics were obtained from a combination of short coded data (eg, Web search snippets) and a larger body of uncoded text, such Wikipedia and Ohsumed or Medline. They saw benefits when the classification tasks were to classify search snippets into different domains (eg, Business, Computers, or Health) and to classify medical abstracts into a small set of disease types (eg, neoplasms, digestive disorders, etc). However, both types of text are relatively homogeneous and do not include dialog or social discourse.

There have also been attempts to classify the dialog acts in online (non-health related) chats. This type of data has some characteristics similar to the breast survivor forums used in this study as the interactions involve peers and ongoing interactions. The main difference between these studies and our work is that the classification of dialog acts addresses the communicative function of an utterance (such as being a greeting, statement, question, or answer), rather than the content; see [24] for a discussion. Annotations based on dialog acts are potentially useful for finding the topic of an information need when the statement of the topic and the expression of need occur in separate sentences or postings. Classification of dialog acts commonly uses a rule-based approach. For example, Wu et al [25] used a search-based procedure to instantiate a set of classification rules, an approach similar to the one we have taken for finding conversations that express a need prior to coding them. In the study reported by Forsyth et al [26], a neural network approach was used. This approach relies on many of the same features as the study reported by Wu et al [25] and achieved an accuracy of 83.2%.

### Conclusions

This research considers the task of identifying the information needs of breast cancer survivors from their postings to online health forums. This approach allows one to assess a broad range of people over a span of years, and to observe true information-seeking rather than self-reports, which can be faulty. We used a supervised classification approach, which is easily repeatable. The sentences within the postings to forums were classified, rather than the posting as a whole, so that we could discriminate among social information, background, and expressions of information need and subsequently identify the general type of the need (such as “medical” or “physical”). Our results show that automatic methods can be an effective method of assessing information needs. One could also perform further processing on the sentences to reveal more specific information, such as names of medications or side-effects.

We also examined whether using expressions of information need to help assess a set of commonly provided education materials was a viable approach. We used well-known methods from information retrieval, mapping sentences onto queries for a search engine with an index of the most frequently provided documents given to patients at the Mayo Breast Clinic. It was found that only a small percentage of information needs are addressed by the provided materials. This finding would explain the use of health forums by breast cancer survivors to seek information as most of their information needs are not easily findable within the brochures they likely received. Further investigation of these unmet needs is warranted to create better materials—and better means of dissemination—in the future. In addition to mobile devices, new opportunities exist for the creation of content that could be delivered by interactive voice assistant products, like Amazon Echo or Alexa or Google Home. It is crucial, however, that to be effective for breast cancer survivors, they must closely target their true information needs.

## Appendix

Multimedia Appendix 1 Annotation Guideline.

Multimedia Appendix 2 Inter-Annotator Agreement.

Multimedia Appendix 3 Comparisons of Classifier Algorithms.

Multimedia Appendix 4 Study of Knowledge Gap Examples.

## Abbreviations

CSN: Cancer Survivor’s Network
CSN-R: data set of randomly selected sentences from the CSN data set
HASN: has information need
MC: MayoConnect
RF: random forest

## References

[1] Boyes A, Girgis A, Lecathelinais C (2009). Brief assessment of adult cancer patients' perceived needs: development and validation of the 34-item Supportive Care Needs Survey (SCNS-SF34). J Eval Clin Pract.

[2] Fong EJ, Cheah WL (2016). Unmet Supportive Care Needs among Breast Cancer Survivors of Community-Based Support Group in Kuching, Sarawak. International Journal of Breast Cancer.

[3] Kwok C, White K (2014). Perceived information needs and social support of Chinese-Australian breast cancer survivors. Support Care Cancer.

[4] Uchida M, Akechi T, Okuyama T, Sagawa R, Nakaguchi T, Endo C, Yamashita H, Toyama T, Furukawa TA (2011). Patients' supportive care needs and psychological distress in advanced breast cancer patients in Japan. Jpn J Clin Oncol.

[5] Park BW, Hwang SY (2012). Unmet needs of breast cancer patients relative to survival duration. Yonsei Med J.

[6] Song H, May A, Vaidhyanathan V, Cramer EM, Owais RW, McRoy S (2013). A two-way text-messaging system answering health questions for low-income pregnant women. Patient Educ Couns.

[7] Vaughan Sarrazin MS, Cram P, Mazur A, Ward M, Reisinger HS (2014). Patient perspectives of dabigatran: analysis of online discussion forums. Patient.

[8] Mulveen R, Hepworth J (2006). An interpretative phenomenological analysis of participation in a pro-anorexia internet site and its relationship with disordered eating. J Health Psychol.

[9] van Uden-Kraan C, Drossaert C, Taal E, Seydel ER, van de Laar M (2008). Self-reported differences in empowerment between lurkers and posters in online patient support groups. J Med Internet Res.

[10] The Haxx Group (2018). Curl:// command line tool and library for transferring data with URLs website.

[11] McRoy S, Jones S, Kurmally A (2016). Toward automated classification of consumers' cancer-related questions with a new taxonomy of expected answer types. Health Informatics J.

[12] Roberts K, Kilicoglu H, Fiszman M, Demner-Fushman D (2014). Automatically classifying question types for consumer health questions. AMIA Annu Symp Proc.

[13] Aronson A (2001). Effective mapping of biomedical text to the UMLS Metathesaurus: the MetaMap program. Proc AMIA Symp.

[14] Hall M, Frank E, Holmes G, Pfahringer B, Reutemann P, Witten I (2009). The Weka data mining software: an update. SIGKDD Explor Newsl.

[15] Blei D, Ng A, Jordan M (2003). Latent Dirichlet Allocation. J Mach Learn Res.

[16] Pennington J, Socher R, Manning C (2014). GloVe: Global Vectors for Word Representation.

[17] Elasticsearch BV (2017). Elastic search.

[18] John L, Andrew M, Fernando P, Brodley C, Danyluk A (2001). Conditional random fields: probabilistic models for segmenting and labeling sequence data. Proceedings of the Eighteenth International Conference on Machine Learning.

[19] Qi L, Chen L, Chen L, Triantafillou P, Seul T (2010). A Linear-Chain CRF-Based Learning Approach for Web Opinion Mining. Web Information Systems Engineering (WISE 2010) Lecture Notes in Computer Science, vol 6488.

[20] Meier A, Lyons EJ, Frydman G, Forlenza M, Rimer BK (2007). How cancer survivors provide support on cancer-related Internet mailing lists. J Med Internet Res.

[21] Zhang T, Cho J, Zhai C (2014). Understanding user intents in online health forums.

[22] Phan X, Nguyen L, Horiguchi S (2008). Learning to classify shortsparse text &amp;amp; web with hidden topics from large-scale data collections.

[23] Roberts K, Killicoglu H, Fiszman M, Demner-Fushman D (2014). Decomposing consumer health questions.

[24] Traum D (2000). 20 Questions on Dialogue Act Taxonomies. Journal of Semantics.

[25] Wu T, Khan F, Fisher T, Shuler L, Pottenger W (2002). Posting act tagging using transformation-based learning. Proceedings of Workshop on Foundations of Data Mining and Discovery.

[26] Forsyth E, Martell C (2007). Lexical discourse analysis of online chat dialog.

