# Supplementary material for: Assessing Unmet Information Needs of Breast Cancer Survivors: Exploratory Study of Online Health Forums Using Text Classification and Retrieval
Source: JMIR Cancer. 2018 May 15;4(1):e10. doi: 10.2196/cancer.9050 (PMC5974460; doi:10.2196/cancer.9050)
Supplement: Multimedia Appendix 1 [file cancer_v4i1e10_app1.pdf]

Multimedia Appendix 1: Breast Cancer Annotation Guideline

Concepts:

We will use 3 columns for coding to capture 2 aspects: the first for Unmet Need and the last two for type(s) of Content.

Every sentence will have at least one, and possibly two types of content. Only a few sentences are explicit expressions of need; the rest should be left blank for that aspect. (Since items may get more than one label, we have eliminated MULT as a type of exception. If there seem to be more than two types, then list the best two.)

We describe the two aspects below.

Expressions of Need:

HASN (Has a need): A question, a request, or a statement about a having mental state (wondering, wanting to know, or not knowing something, being unsure or curious) that involves an explicitly or implicitly mentioned need for some information about breast cancer, beyond an open invitation to talk:

Examples:

Can anyone tell me what ... is like?

Does anyone know ...?

I was wondering <something>

I would like to know <something>

I've been trying to find people who <know about something>

Information Content Types:

SOCL (All Social): Social opening, closings, or commentary. Openings are greetings or generic offers to chat; closings include offering thanks or good wishes for the future. Openings may mention cancer generally, as part of a self introduction or invitation to talk within the BC forum. Commentary might be

something like “That was really helpful” that acknowledges another's contribution.

Examples:

Hi <name>, Hello, I'd be happy to chat"

Thanks, Hang in there, Good luck. God bless. Thank you for your answers. Thank you again.

Anybody out there to talk to about breast cancer?

BACK (Non medical Background): Summarizing one’s non-medical history to provide a context or introduction, for example to justify a concern or establish oneself as able to contribute on various topics. (But descriptions of events that are incidental rather than essential, eg, “I went to the zoo on Friday” or “I bought a dress” should be treated as “other”; see below.)

Examples:

I am retired

MEDI (Medical): Talking (or asking) about medical history, such as mentions of a specific medical diagnosis, test, procedure, treatments, prognosis, or process, including any prescribed drugs, specific treatments, such as mastectomy, Chemotherapy, lumpectomy, radiation, or reconstruction.

Here, a “second opinion” is also classified as a medical thing. However, physical or mental properties that might be observed or perceived such as symptoms or side-effects are covered by another category (PHYS or PSYC).

Examples:

I was diagnosed 4-22-16 with triple negative, 1 lymph node positive. Single Mastectomy 5-31 and I am now going thru round 5 of chemo on 8-31-16 and start 12 weeks in a row after 4 rounds of Doxirubicon.

I would be happy to give you and advice about chemo

PHYS (Physical): Talking (or asking) about physical problems, functionality, or observed side effects of treatment such as hair loss, weight gain, pain, swelling, itchiness or fatigue.

Example:

I was having horrible stiffness in my legs

PSYC (Psychological): Talking (or asking) about issues with mental states such as depression, fear, anxiety, coping, insomnia or physical behaviors that imply mood (eg, crying, laughing, shaking with fear).

Examples:

I became very depressed.

RESO (Information or financial resources): Looking for (or offering help to find) outside sources of information or support, such as special medical providers, books, websites, online forums, support groups, insurance, charities, etc.

Examples:

...the manufacturer sometimes will help ...

...I would Google...

WELL (Wellness): Talking (or asking) about issues with non-medical aspects of wellness including diet, exercise and supplements or foods and drinks (e.g. smoothie, water, vitamins)

Example:

Drinking water really helps.

PREV (Previous topic): Question or statement of interest with a reference that cannot be understood with the previous sentence, that is, it is not clearly about a specific medical treatment or physical symptom/side effect

Examples:

I would like to talk to anyone with the same diagnosis and therapy

Has anyone gone through this to talk to?

One special category should be used for dealing with exceptions:

OTHR (Other information) A statement not covered by any of the other categories. Typical examples of this include: advice that is not wellness (food, exercise) or medical (specific treatment) or resource (book, website); any commentary or judgement that is directed at the self (“I felt stupid”) - but is not about a mental state (such as sadness, anger, etc); descriptions of events that are not part of one’s medical or non-medical history (eg, “I talked to my doctor” or “My doctor talked to me.”)

Below is an example of data coded in a table.

Example:		Unmet need?	Type 1	Type 2 (if any)
a_1	I want to learn about chemotherapy	HASN	MEDI	
a_2	I had a lumpectomy before.		MEDI	
a_3	It was frightening to me.		PSYC	
a_4	I am not sure how successful this might be.	HASN	PREV	

a_5	I called my doctor.		OTHR	
a_6	After the chemo my hair fell out		MEDI	PHYS
