# Supplementary material for: Assessing Unmet Information Needs of Breast Cancer Survivors: Exploratory Study of Online Health Forums Using Text Classification and Retrieval
Source: JMIR Cancer. 2018 May 15;4(1):e10. doi: 10.2196/cancer.9050 (PMC5974460; doi:10.2196/cancer.9050)
Supplement: Multimedia Appendix 2 [file cancer_v4i1e10_app2.pdf]

Multimedia Appendix 2: Inter Annotator Agreement Counts with Percentage Correct based on Final Distribution for each of the data sets

Mayo Connect (MC) data set (1958 sentences)

Category	Annotator 1	Annotator 2	Agreement (% Correct)
Medical	868	601	541 (96%)
Resource	60	87	37 (43%)
Social	236	355	215 (61%)
Psychological	24	61	21 (34%)
Background	23	69	18 (26%)
Wellness	29	88	20 (23%)
Physical	174	168	103 (62%)
Previous	32	147	16 (11%)
Other	413	322	185 (59%)
Multiple	99	60	17 (28%)

Category	Annotator 1	Annotator 2	Agreement (% Correct)
Information need	39	110	22 (20%)
No Information Need	1919	1848	1831 (97%)

Many expressions of information need are indirect. The first version of the annotation guideline was not sufficiently clear regarding these cases and so it was revised after reviewing the ITA results for the MC dataset and before the CSN dataset was annotated.

Cancer Survivors Network (CSN) data set (2246 sentences)

Category	Annotator 1	Annotator 2	Agreement (% Correct)
Medical	389	465	364 (76%)
Resource	95	33	32 (100%)
Social	454	443	384 (87%)
Psychological	74	65	50 (79%)
Background	159	38	34 (89%)
Wellness	86	77	69 (89%)
Physical	161	193	146 (76%)
Previous	734	430	420 (99%)
Other	542	727	454 (62%)

Category	Annotator 1	Annotator 2	Agreement<br>(% Correct)
Information need	159	196	135 (69%)
No Information Need	2087	2048	2025 (99%)

Cancer Survivors Network Random Sentences (CSN-R) data set (1000 sentences)

Category	Annotator 1	Annotator 2	Agreement<br>(% Correct)
Information need	33	30	23 (66%)
No Information Need	967	970	960 (99%)
