# Supplementary material for: Assessing Unmet Information Needs of Breast Cancer Survivors: Exploratory Study of Online Health Forums Using Text Classification and Retrieval
Source: JMIR Cancer. 2018 May 15;4(1):e10. doi: 10.2196/cancer.9050 (PMC5974460; doi:10.2196/cancer.9050)
Supplement: Multimedia Appendix 3 [file cancer_v4i1e10_app3.pdf]

Multimedia Appendix 3: Comparisons of Classifier Algorithms

MA3 Table 1: The performance of a different classifiers on MC data using Naïve Bayes (NB), Support Vector Machines (SVM) and Random Forest (RF) classifiers without local context features.

	**MC data (**without local context features)								
	NB			SVM			RF		
**Category**	**Pre.**	**Rec.**	**F-me.**	**Pre.**	**Rec.**	**F-me.**	**Pre.**	**Rec.**	**F-me.**
**Medical**	0.61	0.60	0.60	0.65	0.65	0.65	0.74	0.73	0.73
**Social**	0.63	0.63	0.63	0.66	0.66	0.66	0.78	0.78	0.78
**Psychological**	0.58	0.59	0.59	0.61	0.61	0.61	0.73	0.72	0.71
**Background**	0.61	0.61	0.61	0.64	0.64	0.64	0.77	0.77	0.77
**Wellness**	0.63	0.63	0.63	0.68	0.68	0.68	0.76	0.75	0.75
**Physical**	0.70	0.68	0.69	0.70	0.70	0.70	0.80	0.79	0.79
**Previous**	0.53	0.53	0.53	0.56	0.56	0.56	0.61	0.61	0.61
**Other**	0.52	0.52	0.52	0.56	0.55	0.55	0.59	0.59	0.59

MA3 Table 2: The performance of a different classifiers on MC data using Naïve Bayes (NB), Support Vector Machines (SVM) and Random Forest (RF) classifiers with local context features.

	**MC data (**with local context features)								
	NB			SVM			RF		
**Category**	**Pre.**	**Rec.**	**F-me.**	**Pre.**	**Rec.**	**F-me.**	**Pre.**	**Rec.**	**F-me.**
**Medical**	0.80	0.80	0.80	0.84	0.84	0.84	0.90	0.91	0.90
**Social**	0.84	0.83	0.83	0.83	0.83	0.83	0.85	0.85	0.84
**Psychological**	0.69	0.70	0.70	0.71	0.71	0.71	0.77	0.75	0.73
**Background**	0.74	0.74	0.74	0.74	0.74	0.74	0.77	0.77	0.77
**Wellness**	0.77	0.77	0.77	0.78	0.78	0.78	0.80	0.79	0.79

**Physical**	0.80	0.78	0.79	0.80	0.80	0.80	0.82	0.83	0.83
**Previous**	0.56	0.56	0.56	0.57	0.56	0.56	0.58	0.58	0.58
**Other**	0.84	0.81	0.82	0.83	0.84	0.83	0.84	0.86	0.85

Supplement Table 3: The performance of a random forest classifier on MC using only words and bigram features, after adding topic modelling, and after adding word embedding features

	**MC data**								
	With only word and bigram features			Adding topic modeling features			Adding word embedding features		
**Category**	**Pre**	**Re**	**F-m**	**Pre**	**Re**	**F-m**	**Pre**	**Re**	**F-m**
**Medical**	0.74	0.73	0.73	0.72	0.71	0.71	0.73	0.72	0.72
**Social**	0.78	0.78	0.78	0.74	0.74	0.74	0.77	0.79	0.77
**Psychological**	0.73	0.72	0.72	0.71	0.71	0.70	0.70	0.71	0.70
**Background**	0.77	0.77	0.77	0.73	0.73	0.73	0.75	0.75	0.75
**Wellness**	0.76	0.75	0.75	0.74	0.73	0.73	0.76	0.75	0.75
**Physical**	0.80	0.79	0.79	0.78	0.78	0.77	0.76	0.76	0.76
**Previous**	0.61	0.61	0.61	0.59	0.59	0.59	0.60	0.60	0.60
**Other**	0.59	0.59	0.59	0.57	0.57	0.57	0.59	0.59	0.59
