# Supplementary material for: Assessing Unmet Information Needs of Breast Cancer Survivors: Exploratory Study of Online Health Forums Using Text Classification and Retrieval
Source: JMIR Cancer. 2018 May 15;4(1):e10. doi: 10.2196/cancer.9050 (PMC5974460; doi:10.2196/cancer.9050)
Supplement: Multimedia Appendix 4 [file cancer_v4i1e10_app4.pdf]

Multimedia Appendix 4: Five examples of queries and the retrieved documents

Post with query	Retrieved document
A query about sensations after mastectomy:<br><br>I have just had a modified rad. mastectomy with a reconstruction. I expect a report from a pathologist anytime. I don't know what my prognosis is at this time. But does anyone know if it is common to have parathesias (or abnormal sensations) immediately after surgery	*A page about scheduling breast MRI*<br>*(It was rated not a match, likely a gap)*<br><br>....cancer and the age at which your relatives developed breast cancer, as well as any precancerous breast lumps you've had. In general, women at average risk of breast cancer should get annual screening mammograms and breast exams beginning at age 40. Women considered at very high risk should generally start getting mammograms and breast MRIs, if recommended, at age 30, according to the American Cancer Society. Because your situation may be different, talk to your doctor about what schedule is best for you. If the results of your screening breast MRI are abnormal, you may need to have more tests done to find out if you have cancer. These are called diagnostic tests.
A query about prognosis<br><br>...Unfortunately, the medical oncologist was not too helpful in the prognosis/ percentage rate of survival". "THAT, I think has been the hardest thing to take". I would really like to know if they think that I won't survive this.	*A page about followup care after cancer treatment:*<br>*(It was rated not a match, likely a gap)*<br><br>Once your cancer treatments are done, talk with your health care provider about future tests you may need. These tests may include physical exams, blood work and X-rays, among others. How often you need appointments depends on your situation. Follow-up visits Talk to your oncologist about your follow-up appointments. Find out the care provider to see and who to contact if you have questions or concerns. In most cases, you will see your primary health care provider for your regular and preventive medical care. Depending on your situation, you will likely see your oncologist for care that relates to your cancer treatment. What you can do Take these steps to get ready for your appointments: Write down any symptoms you have. Make a list of all medications, vitamins and supplements that you take. Ask a family member or friend to come with you, if possible. Sometimes it can be hard to remember all you are told during an appointment. Someone who comes with you may remember something that you missed or forgot. Write down questions to ask your provider. In addition to the questions you prepare, ask questions during your appointment any time you don't understand something.
A query about supporting a friend who has cancer<br><br>Is there anything I can do to encourage her to grieve. Is it better that she doesn't. I'm 29 and she's 51. "We've never experienced this before, she's never had it in her family". I'm really trying to find some support so I can support her at this	*A page about relationships and support for coping with breast cancer.*<br>*(It was rated a match, therefore no gap.)*<br><br>Strong relationships can be crucial in coping with breast cancer. Friends and family are often a central part of your treatment, but other kinds of support can be helpful as well. Consider meeting with a counselor, medical social worker or clergy member. Do not think that you have to confront your cancer alone. Counselors, chaplains, psychologists and psychiatrists are available at Mayo Clinic to help people with cancer cope during this difficult experience. Tell your health care provider if you would like to speak with a counselor. You may find the concern and understanding of other women with breast cancer especially comforting. Breast cancer survivors have developed a tremendous support network. For example, the Reach to Recovery program coordinated by the American Cancer Society puts a woman who has breast cancer in contact with a volunteer who also has

time.	experienced breast cancer. The volunteer can provide information and support from the time you receive your diagnosis and throughout your treatment and recovery. Maintaining a positive outlook Breast cancer affects your life and the lives of those closest to you. It can create fear and uncertainty and may sometimes strain relationships. It also may change you physically and emotionally. And, too often, the emphasis on treating your disease takes priority over healing you as a person. Both are crucial to your recovery. It can be helpful to know that some changes breast cancer creates in your life can be positive. Many breast cancer survivors find that their lives and relationships become deeper and more meaningful. Take time to examine what is most important to you. Think about the things you want to accomplish and how you can achieve your goals. Give priority to doing things you most enjoy and living your life to the fullest.
A query about weight gain after Tamoxifen:<br><br>I have been on Tamoxifen for a year now and have gained so much weight. Has anyone else had this problem	*A page about role of hormones and the use of Tamoxifen in treatments:*<br>*(It was rated a match, therefore no gap.)*<br><br>Normal breast tissue cells have receptors that receive the female hormones estrogen and progesterone. The receptors allow the cells to use these hormones to grow. Many breast cancer cells also have the same receptors. When they do, they are said to be hormone receptor positive. They are estrogen receptor positive or progesterone receptor positive or both. Hormonal therapy uses medication that travels through your body to either reduce the amount of hormones that reach hormone-sensitive cancer cells or block the action of estrogen so cancer cells stop growing and die. If a piece of your tumor is removed, biopsied, it can be evaluated to determine whether it is estrogen receptor positive. If it is positive for either receptor, you may benefit from hormonal therapy, which lessens the chance that breast cancer will return in the breasts or elsewhere in the body. If your cancer is not estrogen or progesterone positive, hormonal therapy will not be recommended. Women whose tumors are hormone receptor positive generally have a lower rate of cancer returning in that location and have a longer overall survival rate. Hormonal therapy appears to lessen the chance of recurrence and improve survival rates. Tamoxifen Tamoxifen (Nolvadex) blocks a tumors ability to use hormones. It typically is taken daily in pill form for five years after cancer surgery. Tamoxifen is available for women who are premenopausal or postmenopausal. Common side effects of tamoxifen include: Hot flashes or night sweats. Nausea. Irregular periods in women prior to menopause. Vaginal discharge. Vaginal itching, dryness or irritation. Less frequent symptoms include depression, loss of appetite, eye problems such as cataracts, headache, and weight gain. There also can be an increased risk of blood clots, strokes and uterine cancers such as endometrial cancer.
A query about regrowth of hair<br><br>I finish my course of chemotherapy 4 Chemotherapy medication followed by 4 taxol on March 15. When should I expect my hair to really start growing	*A page about the purpose and side effects of chemotherapy.*<br>*(It was not rated as a match, but it comes close.)*<br><br>Chemotherapy may lessen the risk of your cancer returning or spreading. However, chemotherapy affects healthy cells especially fast-growing cells in your digestive tract, hair and bone marrow as well as cancerous ones. Due to the variety of cells affected, chemotherapy can cause many side effects. Short-term side effects include: Digestive tract effects such as appetite change, weight change, mouth sores, sore throat, nausea, vomiting, diarrhea and constipation. Bone marrow-related effects such as fatigue, bruising, bleeding, decreased white blood cell counts, and susceptibility to infections. Hair loss. Although temporary, hair loss can be one of the most difficult side effects of chemotherapy. Ask the members of your health care team for resources to help you cope with hair loss. Long-term risks include: Potential loss of ovarian function which can lead to infertility or early menopause. Small risk of side effects on the heart such as weakening of the heart muscle. Small risk of getting other cancers such as leukemia. Side effects vary depending on the medications used. Side effects are often more severe with higher doses of medications. They may get worse during the course of treatment. If the number of white cells in your blood drops, chemotherapy may be stopped until your white cells return to a safe level. Not everyone has side effects. But, there are ways to help control side effects if you suffer from

	them. Taking other medications may help relieve some symptoms such as nausea. Relaxation techniques such as meditation and deep breathing may help reduce stress. See Complementary medicine on page 31. In addition, exercise has been shown to help lessen fatigue caused by chemotherapy. Wearing wigs, hats or turbans can make hair loss less obvious. While having chemotherapy, most people are able to continue their normal activities for all but a day or two a month during their treatment months. Most side effects resolve when treatment ends.
